# Elevated plasma oxytocin levels and higher satisfaction with life in young oral contraceptive users

**DOI:** 10.1038/s41598-020-64528-w

**Published:** 2020-05-19

**Authors:** Benjamin Garforth, Helle Degnbol, Elizabeth T. Terris, Paul J. Zak, Michael Winterdahl

**Affiliations:** 1 Department of Nuclear Medicine and PET Centre, Aarhus University, Aarhus, Denmark; 2 Translational Neuropsychiatry Unit, Aarhus University, Aarhus, Denmark; 3 Center for Neuroeconomics Studies, Claremont Graduate University, Claremont, CA USA

**Keywords:** Neuroscience, Endocrinology

## Abstract

Oral contraception (OC) is used by approximately fifty-five million women in the USA alone and is listed as an essential medicine by the World Health Organisation. Altered mood is a common reason for OC cessation. Here we investigate the effects of OC on hormones that are linked to mood. We obtained blood samples from 185 young women (average age 21.2) in two cohorts and tested the effects of OC on plasma levels of oxytocin, adrenocorticotropic hormone (ACTH), estradiol, progesterone and testosterone. We related plasma hormone levels with self-reported measures of mood, well-being and depression. OC-users in both cohorts showed elevated basal oxytocin, lower ACTH, estradiol, progesterone and testosterone compared with non-OC users. Satisfaction With Life Score (SWLS) was higher in OC -users compared to non-OC users, with no differences in the Beck Depression Score (BDI) and Positive And Negative Affect Schedule (PANES). In conclusion, our data show alterations in hormone levels and SWLS in response to OC.

## Introduction

In the United States, 98% of sexually active women have used birth control at some point in their lives, and 62% of women of reproductive age currently use birth control^1^. Overwhelmingly, birth control is managed with oral contraceptives (OC). OC are recognised as an essential medication by the World Health Organisation and are critical for family planning and to protect women’s health^2^. Despite this, OC can have adverse effects on mood^3,4^. These mood effects cause many women to discontinue OC use.

Hormones, particularly those that circulate in the brain, can have profound effects on mood^5^. Foremost among these are stress hormones, including adrenocorticotropin hormone (ACTH) and cortisol^6^. Gonadal hormones, including estrogen and progesterone, as well as oxytocin, also affect mood, particularly in women^7^. Oxytocin is of particular interest because it has been shown to reduce anxiety through down-regulation of the hypothalamic–pituitary–adrenal (HPA) axis^8–10^, though whether those with anxiety disorders have higher or lower basal oxytocin has not been established^11,12^. In addition, estrogen receptor β (ERβ) activation reduces anxiety-related behaviours^13^ and acts as a transcription factor for oxytocin^14^, suggesting a role for oxytocin in ERβ-mediated anxiolytic effects.

Women suffer from depression from 50% to 100% more often than men^15^. This discrepancy is partially due to hormonal differences between the sexes^16^. Women are particularly susceptible to depressive symptoms when estrogen levels are low, including premenstually^17^ and during menopause^18^. The most potent estrogen, estradiol, has been shown to have strong anxiolytic and antidepressant effects in humans and has been used to alleviate symptoms of menopause^19^. However, the effects of prolonged exposure to high estradiol levels on mood, as seen in OC -users, is less well characterised, though is found to correlate with the risk of depression in males^20^. Rates of depression vary with the type of OC used and particular individuals may be more susceptible to depression during OC use^21^. A study of all women of reproductive age in Denmark (N = 106,199) found that OC increased the relative risk of depression, compared to non-OC users, from between 1.23 to 2.0 depending on the type of OC used^22^.

The aim of the present study was to investigate plasma levels of oxytocin, stress and sex hormones, as well as self-reported measures of mood and well-being, in OC -users compared to non-users in order understand the impact of OC use.

## Results

**Figure 1:**
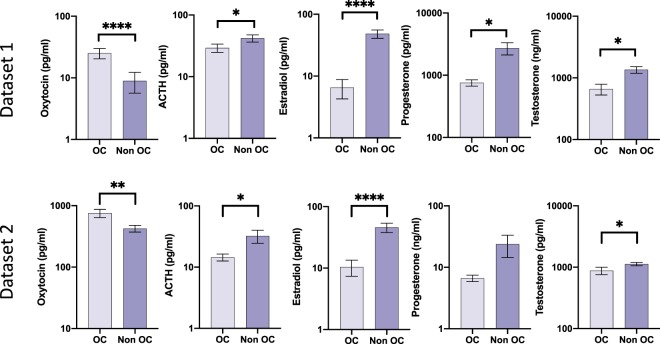
Graphs showing associations between OC use and hormone levels. Dataset1: Oxytocin (n = 129), ACTH (n = 136), Estradiol (n = 56), Progesterone (n = 73), Testosterone (n = 94). Dataset 2: Oxytocin (n = 47), ACTH (n = 38), Estradiol (n = 49), Progesterone (n = 48), and Testosterone (n = 47). *indicates significance at p < 0.05, **indicates significance at p < 0.01, ****indicates significance at p < 0.0001.

In dataset 1, there was higher plasma oxytocin in OC-users (1.1 ± 0.5) compared to non-users (0.6 ± 0.5); t(127) = 5.33, p < 0.001, lower ACTH in OC-users (1.3 ± 0.4) compared to non-users (1.5 ± 0.4); t(134) = 2.33, p = 0.021, lower estradiol in OC-users (0.8 ± 0.3) compared to non-users (1.5 ± 0.5); t(54) = 5.48, p < 0.001, lower progesterone in OC-users (0.2 ± 0.1) compared to non-users (0.4 ± 0.3); t(71) = 2.58, p = 0.012 and lower testosterone in OC-users (0.2 ± 0.1) compared to non-users (0.3 ± 0.2); t(92) = 3.50, p < 0 .001. In dataset 2, there was higher plasma oxytocin in OC-users (2.8 ± 0.3) compared to non-users (2.6 ± 0.2); t(45) = 3.27, p = 0.002, lower ACTH in OC-users (1.1 ± 0.2) compared to non-users (1.3 ± 0.4); t(36) = 2.19, p = 0.035, lower estradiol in OC-users (0.7 ± 0.5) compared to non-users (1.5 ± 0.4); t(47) = 6.12, p < 0.001, and lower testosterone in OC-users (2.9 ± 0.2) compared to non-users (3.0 ± 0.1); t(45) = 2.82, p = 0.007. No significant differences were observed in progesterone levels in OC-users (0.8 ± 0.3) compared to non-users (0.9 ± 0.5); t(46) = 1.55, p = 0.127. Figure 1 shows the associations between OC use and hormone levels.

Psychometric measures are summarised in Table 1. Higher satisfaction with life (Satisfaction With Life Scale, SWLS) was observed in OC-users compared to non-users in both datasets; with no significant difference in positive or negative affect (Positive and Negative Affect Schedule, PANAS, dataset 1) or depressive symptoms (Beck Depression Inventory, BDI, dataset 2). In datasets 1 and 2, respectively, 55.1% and 59.1% of OC-users were in a relationship compared to only 35.6% and 29.6% of non-users. In dataset 1, OC-users reported being more sexually active and having a higher number of sexual partners compared to non-users. A correlation between being in a relationship and sexual activity was observed, Pearson’s r (129) = 0.50, p < 0.001. A similar relationship was observed in dataset 2, Pearson’s r (47) = 0.68, p < 0 .001.

**Table 1:** Comparison of OC -users and non-users from both studies.

Dataset 1							Dataset 2						
		Oral contraceptive users	Non-users	statistics	df	p			Oral contraceptive users	Non-users	statistics	df	p
**Number of Participants**		49	87	—	—	—	**Number of Participants**		22	27	—	—	—
**Age (years)**		20.7 ± 4.9	21.0 ± 4.6	t = 0.3	134	0.79	**Age (years)**		22.2 ± 5.4	22.1 ± 6.3	t = 0.0	46	0.98
**Weight (kg)**		61.5 ± 8.2	61.1 ± 13.5	t = -0.3	133	0.80	**Weight (kg)**		57.6 ± 14.3	61.6 ± 10.0	t = 1.1	47	0.28
**Last menstruation** **Current,** **Last week,** **2 weeks ago,** **3-4 weeks ago,** **More than 4 weeks ago** **Don't remember**		11 (22.4%) 13 (26.5%) 8 (16.2%) 8 (16.2%) 8 (16.2%) 1 (2%)	17 (12.5%) 17 (12.5%) 14 (10.3%) 28 (20.6%) 7 (5.1%) 4 (2.9%)										
**Relationship**	**yes**	27 (20.5%)	31 (23.5%)				**Relationship**	**yes**	13 (26.5%)	8 (16.3%)			
	**no**	18 (13.6%)	56 (42.4%)	χ^2^ = 7.6	1	0.007*		**no**	9 (18.4%)	19 (38.8%)	χ^2^ = 4.3	1	0.04*
**Sexually active**	**yes**	27 (20.3%)	22 (16.5%)				**Sexually active**	**yes**	14 (28.6%)	11 (22.4%)			
	**no**	22 (16.5%)	62 (46.6%)	χ^2^ = 11.1	1	<0.001*		**no**	8 (18.3%)	16 (32.7%)	χ^2^ = 2.5	1	0.11
**Sex/month**		5.4 ± 8.4	2.0 ± 4.9	t = -3.0	132	0.004*	**Sex/month**		8.6 ± 8.6	4.7 ± 8.6	t = -1.5	47	0.13
**Number of sexual partners**		2.8 ± 3.8	1.7 ± 2.5	t = -2.1	133	0.04*	**Number of sexual partners**		4.2 ± 5.4	2.5 ± 3.2	t = -1.3	47	0.19
**Number of close friends**		8.1 ± 6.8	7.4 ± 4.5	t = -0.7	132	0.49	**Number of close friends**		8.3 ± 5.6	6.6 ± 2.5	t = -1.4	47	0.16
**Alcohol/month**		7.3 ± 10.4	6.3 ± 9.9	t = -0.5	133	0.60	**Alcohol/month**		21.4 ± 15.4	11.5 ± 14.1	t = -2.2	43	0.03*
**Have you ever used** **recreational drugs?**	**yes**	17 (13.3%)	34 (26.6%)				**Have you ever used** **recreational drugs?**	**yes**	12 (24%)	13 (26.5%)			
	**no**	24 (18.8%)	53 (41.4%)	χ^2^ = 0.07	1	0.80		**no**	10 (20.4%)	14 (28.6%)	χ^2^ = 0.2	1	0.66
**SWLS**		26.9 ± 5.3	24.8 ± 6.1	t = -2.0	133	0.04*	**SWLS**		29.5 ± 4.5	24.5 ± 6.5	t = -2.9	44	0.006*
**PANAS positive**		2.8 ± 0.8	2.9 ± 0.7	t = 0.8	134	0.44	**BDI**		24.2 ± 3.9	27.1 ± 7.0	t = 1.7	47	0.09
**PANAS negative**		1.4 ± 0.3	1.4 ± 0.4	t = 0.3	134	0.77							

Relationship does not distinguish between partner gender. Number of sexual partners and recreational drugs refers to the last five years. SWLS refers to the Satisfaction With Life Scale, PANAS to The Positive and Negative Affect Scale, and BDI to the The Beck Depression Inventory. All values are given as mean plus and minus standard deviation. Varying degrees of freedom is due to not all questions being answered by the participants.

**Figure 2:**
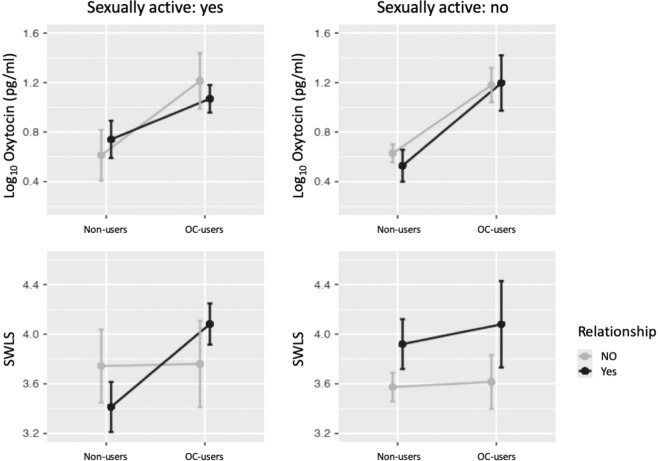
Log_10_ transformed oxytocin levels (top) and SWLS (bottom) for oral contraceptive (OC) users and non-user divided by sexual activity and relationship status. No effect of sexual activity or relationship status on oxytocin levels were found whereas sexually active OC -users in a relationship have significantly higher SWLS than sexually active non-users in a relationship.

Both relationship status and sexual activity could affect the secretion of oxytocin and SWLS. Estimating a general linear model of oxytocin levels, OC-use, relationship status, and their interactions applied to the larger of the two datasets, dataset 1, revealed a significant main effect of OC-use, F(121,3)  =  31.1, p  < 0 .001. There were no significant main or interaction effects of relationship status. As we observed a high correlation between being in a relationship and sexual activity, the latter could not be included in the model. Figure 2 illustrates the relationship between oxytocin, OC-use, relationship status and sexual activity. General linear model estimates of OC-use and relationship status on SWLS and their interactions for the same data failed to reveal significant main or interaction effects of OC-use or relationship status. Figure 2 shows that the largest difference in SWLS between OC -users and non-users is found in sexually active women in a relationship. No effect of recreational drug use on SWLS was observed.

## Discussion

We showed that basal oxytocin levels are significantly higher in OC -users compared to non-users. Compared to estradiol, the active estrogen component in most combined oral contraceptive pill formulations, ethinylestradiol, has similar affinities to estradiol receptors and a longer half-life^23,24^. Estrogen receptor beta (ERβ) is a transcription factor for the oxytocin gene^14^ and we posit that sustained signalling could result in the increased oxytocin expression observed. The G-Protein Coupled Estrogen Receptor (GPER1 or GPR30) accounts for the rapid effects of estradiol signalling. Acute treatment of hypothalamic cells with estradiol *in vitro* has been shown to induce rapid exocytosis of oxytocin^25^. Oxytocin has been shown to reduce anxiety through its down-regulation of the hypothalamic–pituitary–adrenal axis^8,9^.

Our findings are consistent with early research by Silber and co-workers^26^, as well as studies of oxytocin levels in peripartum and postpartum periods, indicating a relationship between altered oxytocin levels and depressive symptom severity^27,28^. However, the analysis of both datasets used in the current study showed higher SWLS in OC -users compared to non-users and no significant difference in affect or depressive symptoms. While SWLS negatively correlates with BDI, both can in principle be used as measures of well-being^29^. However, it is worth noting that clinically depressed subjects were excluded from the present study and Fig. 2 indicates that the differences in SWLS are driven by a complex interplay between relationship status, sexual activity, and OC use.

Previous research has suggested that OC may provide a mood stabilising effect^3^. Jarva and Oinonen found that OC -users experienced a blunted positive affective response to tasks when compared with non-users and men whereas the groups did not differ in terms of negative affect reactivity^30^. A blinded and randomized trial of 3 months of OC (150 μg levonorgestrel and 30 μg ethinylestradiol) use or placebo for 332 women reported decreased well-being in the OC group^31^. Unlike these findings, PANAS scores did not differ between groups in our study. Downregulation of the oxytocin receptor or decreased oxytocin secretion following e.g. a social cue are however plausible consequences of chronic, elevated oxytocin levels and we hypothesize that a higher baseline oxytocin level results in proportionately lower activity of the oxytocinergic system and therefore reduced positive affect. However, the higher SWLS in OC -users compared to non-users could also be due to survivorship bias whereby OC -users who suffer negative symptoms terminate OC use and the women who have no negative symptoms or experience beneficial effects continue use^3,32^. This results in a sampling bias, also noted by Skovlund and co-workers^22^ that may account for previous studies that found no link between OC use and depression.

ACTH levels were significantly lower in OC -users, consistent with previous work demonstrating alterations to the HPA axis^32^. Our finding of lower levels of estradiol, progesterone, and testosterone among OC -users are also in line with current literature^33–35^. Although OC are often perceived as a homogenous group of drugs, this is not the case. Older formulations of OC that either contain ethinylestradiol (such as Levonorgestrel-ethinylestradiol) or progesterone-only pills (Levonorgestrel only) activate negative feedback mechanisms to decrease sex hormone production but will not be detected by standard serological assays resulting in a reported decrease in sex hormones consistent with our findings. More recently, bioidentical estradiol combined oral contraceptive pill formulations have been approved, such as Dienogest-estradiol valerate pills in 2009 and Nomegestrol acetate-estradiol in 2010. In both cases, negative feedback from ethinylestradiol or bioidentical estradiol will result in decreased production of endogenous estradiol and lower sex hormone levels. However, only the more recent estrogen derivatives are converted to estradiol *in vivo* and form an exogenous supply of estradiol that can be detected along with endogenous estradiol. Thus, future studies may not find lower levels of estradiol in OC -users. Both progestins and ethinylestradiol are known to have potent antiandrogenic activity^33,36,37^, resulting in decreased testosterone production and increased production of the inactivating protein sex hormone binding globulin. In the present study we measured total testosterone levels. Free testosterone levels typically parallel total testosterone levels. However, treatment with estrogens, as seen in OC -users, can change sex hormone binding globulin (SHBG) levels as well as the availability of testosterone binding sites. Thus, both free and total testosterone levels should be evaluated in future studies^38^. Ethinylestradiol is unable to bind to SHBG and the total serum concentration is bioavailable^39^. Similarly, progestins have an unusually high percentage of unbound form in the blood^40^. Therefore, SHBG and corticosterone-binding globulin is assumed to have minimal impact on the signaling efficacy of ethinyl-estradiol and progestins.

The lack of information about the duration and the specific OC formulations used by our subjects is a major limitation of the present study and future studies regarding the effect of specific OC formulations on hormone levels are needed. Reliable measurements of hormone levels may as well be a limiting factor in this analysis, especially for oxytocin. The enzyme-linked immunosorbent assay (ELISA) method for detecting oxytocin, used in dataset 2, is less precise compared to radioimmunoassay (RIA) with an extraction step as the products of oxytocin degradation are likely to contribute to reported oxytocin levels^41^. Although the different methods of oxytocin measurement did not allow us to merge the two datasets, oxytocin levels were consistently higher in OC -users in both datasets.

Participants’ menstrual cycles may have also affected our results. Peripheral oxytocin levels increase during the menstrual cycle until ovulation and then decrease until menstruation when the cycle begins again^42,43^. The magnitude of this variation in oxytocin differs widely between women^44^, making it difficult to generalise a “baseline” oxytocin level in freely cycling women for comparison to OC -users. As noted by Salonia and co-workers^45^, women using OC do not show this variation. Few freely-cycling participants were ovulating based on self-reports and therefore unlikely to have significantly impacted the mean oxytocin levels in our non-OC user dataset.

In conclusion, we have shown higher oxytocin levels and higher SWLS in OC -users compared to non-users. We posit that OC increases plasma oxytocin levels, thereby stabilising mood and blunting positive affect.

## Methods

We analysed the relationship between OC use and hormones using two previously published datasets^46,47^. Both studies were similar methodologically and the current study includes only women who identified their birth control method. A brief description is included below.

Dataset 1: Participants were recruited from Claremont Graduate University, Westmont College and local organisations within the Claremont, CA and Santa Barbara, CA communities. Three hundred and ninety-nine females participated, of which 136 reported birth control practice and were included in the current analysis.

Dataset 2: Participants were recruited from Claremont Graduate University and Scripps College. Sixty females participated, of which 49 reported birth control method.

All participants were at least 18 years of age and were screened by a clinical psychologist 2–4 weeks prior to the study for depression and other severe comorbid psychopathologies. On the morning of the study, demographic information, OC use and psychological questionnaires were acquired using a computerized survey, after written consent, but immediately prior to the blood draw. This was done to minimize human interactions that might affect plasma oxytocin levels. Blood samples in both studies were obtained by a qualified phlebotomist. Twelve ml of blood were drawn from the antecubital vein with an EDTA whole-blood tube using a Vacutainer blood collection kit. The tubes were rocked to prevent coagulation and ensure mixing, before being placed on ice. Within 15 minutes, the tubes were centrifuged at 1500 rpm for 12 minutes at 4 °C. The supernatant was extracted and stored in 2 ml microtubes at −80 °C until analysis.

Samples for dataset 1 were analysed at the Reproductive Endocrine Research Laboratory at the University of Southern California, Los Angeles, CA. Oxytocin was assayed from plasma using a RIA kit produced by Bachem, Torrance, CA. The oxytocin assay included an extraction step to reduce binding of side products^48^. Remaining hormones were assayed by Yerkes National Primate Research Center at Emory University, Atlanta, Georgia, using commercial RIA and ELISA kits from DiaSorin, Inc., Stillwater, MN (ACTH), Diagnostic Systems Laboratories, Webster, TX (estradiol), Siemens, Los Angeles, CA (progesterone), Beckman Coulter, Webster, TX (testosterone), The inter- and intra-assay coefficients of variation were less than 4% (OT) and 15% (remaining hormones).

Samples from dataset 2 were analysed at the Endocrine Core Laboratory of the Yerkes National Primate Research Center at Emory University, Atlanta, Georgia, USA. Commercial RIA and ELISA kits from Assay Designs, Ann Arbor, MI (oxytocin), DiaSorin Inc., Stillwater, MN (ACTH), Diagnostic Systems Laboratories, Webster, TX (estradiol), Siemens, Los Angeles, CA (progesterone), and Beckman Coulter, Webster, TX (testosterone). All inter-assay and intra-assay coefficients of variation were within acceptable bounds (<15%).

Participants completed psychological questionnaires with self-reported measures. Overall mood state and well-being were measured using the Satisfaction With Life Scale (SWLS)^29^ that assesses the respondent’s judgement of life satisfaction. The SWLS questionnaire consists of five statements, such as”In most ways my life is close to my ideal”, which are rated on a 1–5 point scale, where 1 is “strongly disagree” and 5 is “strongly agree”. A high score indicates great satisfaction with life, while a low score indicates dissatisfaction.

The Positive and Negative Affect Scale (PANAS)^49^ contains 20 adjectives that describe affective states (10 items for negative affect and 10 items for positive affect) that participants rate “at this moment” on a Likert scale ranging from 1 (very slightly or not at all) to 5 (extremely).

The Beck Depression Inventory (BDI)^50^ is a 21-item survey assessing an array of symptom categories, including mood, pessimism, a sense of failure, a lack of satisfaction, and self-hatred. Each item is scored 0 to 3. An aggregate score below 13 represents no or minimal depression; scores from 14–19 represent mild depression; scores of 20–28 indicate moderate depression; and scores of 29 or more show severe depression. The BDI was used to screen participants already suffering from clinical depression; these individuals were excluded from this analysis.

All participants gave written informed consent in accordance with the Helsinki Declaration of 1975, revised in 2008. The protocol was approved by the institutional review boards at Claremont Graduate University, Westmont College, and Scripps College.

### Statistics

Hormone concentrations were log transformed as a normalization prior to analysis. Log transformed concentrations are given without units. Student’s t-tests were used to compare psychological measures and log-transformed hormone data between OC-users and non-users; no correction for multiple comparison was performed. Correlations between relationship status and sexual activity were computed by Pearson product moment correlations; no covariates were included in the analysis. Possible effects of relationship status and sexual activity on levels of oxytocin and SWLS where explored using a generalized linear model. For all analyses, the significance level was set at 0.05. Results are expressed as mean ± the standard deviation (SD).

## Data Availability

The datasets generated during and/or analysed during the current study are available from the corresponding author on request.
